# Patients with Diverticular Disease Have Different Dietary Habits Compared to Control Subjects: Results from an Observational Italian Study

**DOI:** 10.3390/nu15092119

**Published:** 2023-04-28

**Authors:** Barbara Polese, Marilia Carabotti, Sara Rurgo, Camilla Ritieni, Giovanni Sarnelli, Giovanni Barbara, Fabio Pace, Rosario Cuomo, Bruno Annibale, on behalf of REMAD Group

**Affiliations:** 1 Department of Clinical Medicine and Surgery, University of Naples Federico II, 80131 Naples, Italy; barbara.polese@gmail.com (B.P.); sara.rurgo@unina.it (S.R.); sarnelli@unina.it (G.S.); 2 Department of Medical-Surgical Sciences and Translational Medicine, Sapienza University, 00189 Rome, Italy; camilla.ritieni@gmail.com (C.R.); bruno.annibale@uniroma1.it (B.A.); 3 Department of Medical and Surgical Sciences, University of Bologna, 40138 Bologna, Italy; giovanni.barbara@unibo.it; 4 Complex Operative Unit (UOC) of Gastroenterology, Bolognini Hospital, 24068 Seriate, Italy; fabio.pace@unimi.it; 5 UOC of Gastroenterology, AORN Sant’Anna e San Sebastiano, 81100 Caserta, Italy; rcuomo67@gmail.com

**Keywords:** diet surveys, dietary habits, diverticulosis, colonic, diverticulitis, colonic, dietary fats, dietary proteins, dietary fibre, vitamins

## Abstract

The role of dietary habits as risk factor for the development of diverticular complications has strongly emerged in the last years. We aimed to evaluate possible differences in dietary habits between patients with diverticular disease (DD) and matched controls without diverticula. Dietary habits were obtained from standardized food frequency questionnaires collected at entry to the Diverticular Disease Registry (REMAD). We compared controls (C) (*n* = 119) with asymptomatic diverticulosis (D) (*n* = 344), symptomatic uncomplicated diverticular disease (SUDD) (*n* = 154) and previous diverticulitis (PD) (*n* = 83) patients, in terms of daily calories, macro and micronutrients and dietary vitamins. Daily kcal intake and lipids, both saturated and unsaturated, were significantly lower in patients with DD than C. Total protein consumption was lower in PD than D, with differing consumption of unprocessed red meat, white meat and eggs between groups. Consumption of fibre, both soluble and insoluble, was lower in patients with PD compared to patients with SUDD, D and C, whereas dietary vitamins A, C, D and E and Oxygen Radical Adsorbance Capacity index were lower in all DD groups compared to C. This observational study showed that DD patients have different dietary habits, mainly in terms of caloric, fat, fibre and vitamin intake, compared to control subjects.

## 1. Introduction

Diverticular disease (DD) represents a common condition in Western countries, being the fifth-most costly gastrointestinal disorder considering direct and indirect costs in the United States [1]. Prevalence of colonic diverticula increases with age, affecting up to 65% of people older than 80 years [2]. Most people with colonic diverticula remain asymptomatic for life (diverticulosis), but about 15–20% experience abdominal symptoms (e.g., abdominal pain, changes in bowel habits and/or bloating), a condition called symptomatic uncomplicated diverticular disease (SUDD), and a smaller proportion of patients may develop diverticular complications, such as acute diverticulitis or diverticular bleeding [3]. In recent years an increasing trend towards complicated disease has been reported, especially in Western countries [4,5,6,7]. Therefore, in relation to its epidemiologic and economic burden, it is matter of interest to assess the presence of risk or protective factors able to prevent symptomatic DD and/or diverticular complications. 

At this time, one of the most interesting topics, both for physicians and patients, is inherent to dietary habits. Most available data have focused on the role of fibre intake, Western dietary patterns and the risk of diverticular complications. In particular, large prospective women cohort studies have found that higher fibre intake is associated with reduced risk of diverticular disease and diverticulitis, with a protective effect for cereals and fruit fibre, but not for vegetables [8,9]. Compared to women who consume less than 18 g/day total fibre, those consuming almost 25 g/day of fibre have a 13% reduced risk of incident diverticulitis (HR 0.87, 95% CI: 0.79–0.96) [8]. In addition, two other large prospective studies have shown an association between red meat consumption, in particular unprocessed red meat, and increased risk of diverticulitis [10,11]. One of those studies focuses on differentiating Western dietary patterns (defined as high in red meat, refined grains and high-fat dairy), and prudent dietary patterns (defined as high in fruits, vegetables and whole grains). Compared with those who have a prudent dietary pattern, people with a Western dietary pattern have a 1.55 times higher risk of acute diverticulitis (HR 1.55, 95% CI: 1.20–1.99) [10]. 

However, to our knowledge, no study provided a comparison between different stages of diverticular disease (diverticulosis, SUDD and previous diverticulitis) in control subjects without diverticula, nor assessed separate possible risk or protective dietary factors for diverticulosis, SUDD or complicated disease. In addition, the role of dietary factors different from fibre and meat in diverticular disease has been scarcely investigated. In particular, the possible role of low levels of circulating vitamin D has been associated with diverticulitis [12], but no data regarding dietary vitamin D or dietary antioxidant vitamins (vitamin A, C and E) are available.

The aim of this study was to evaluate possible differences in dietary habits between controls without diverticula, asymptomatic diverticulosis or SUDD and previous diverticulitis patients, in terms of daily calories, macro- and micronutrients and dietary vitamins.

## 2. Materials and Methods

### 2.1. Study Design

Data were obtained from the Diverticular Disease Registry (REMAD), promoted by the Italian Study Group on Diverticular Disease (GrIMAD). As previously reported, the REMAD registry is a 5-year prospective, observational, multicentre cohort study, primarily aimed at investigating the natural history of asymptomatic diverticulosis and diverticular disease [13,14,15]. Study methods were extensively reported in previous publications [13,14,15]. Briefly, 1217 patients with DD were consecutively enrolled by 47 Italian centres during the two-month recruitment period. Of these centres, 23 adhered to the nutritional survey. The present study focuses on the baseline dietary data collected during the nutritional survey. Inclusion criteria were informed consent, age ≥ 18 years and endoscopic/radiologically-confirmed colonic diverticula. Exclusion criteria were failure to sign informed consent and inability to adhere to the study procedures. A group of asymptomatic subjects matched for age, gender and BMI, without evidence of colonic diverticula and who underwent a colonoscopy for colorectal cancer screening served as controls. 

### 2.2. Data Collection

At the study entry, patients were categorised into four subgroups according to the following criteria:

(a) Controls (C): asymptomatic for upper and lower gastrointestinal symptoms, without endoscopic evidence of colonic diverticula or other organic disease (colonoscopy performed within two years);
(b) Diverticulosis patients (D): with presence of colonic diverticula in the absence of abdominal symptoms;
(c) SUDD patients: with recurrent abdominal pain, mainly in the lower abdominal quadrants, with a frequency of at least once weekly, present for at least six months, and/or changes in bowel habit, without a well-defined previous attack of acute diverticulitis;
(d) Previous diverticulitis patients (PD): with at least one past episode of acute diverticulitis, complicated or not.

All patients filled out a standardized food frequency questionnaire (FFQ) [16] that investigated the consumption of 29 items of food [pasta/rice, potatoes, bread, crackers/rusks, dried legumes, canned legumes, red meat, white meat, fish, eggs, milk, yogurt, aged cheeses, fresh cheeses, ham/speck/bresaola, cured meats, raw vegetables, cooked vegetables, fruit, cookies, sweet snacks/sweets, salty snacks, fruit juices, sugary drinks, wine, beer, spirits, animal-based condiments (e.g., butter, lard, bacon, cream), vegetable condiments (e.g., olive oil, margarine)] referring to the dietary habits of the previous three months. For each food, the FFQ gathered data about the size portions (‘small’, ‘medium’ and ‘large’) and frequency of assumption (‘more than once a day’, ‘once a day’, ‘more than once a week’, ‘once a week’, ‘more than once a month’, ‘rarely/never’). 

The estimation of caloric, macronutrient and micronutrient daily intake was performed using a specific software (Winfood, Medimatica Srl Unipersonale, Colonnella [TE], Italia) that provides bromatological analysis based on an Italian food composition database [17]. In detail, we assessed and compared daily consumption of calories (kcal/day), lipids (saturated fatty acids, monounsaturated fatty acids and polyunsaturated fatty acids expressed as g/day), proteins (expressed as g/day), carbohydrates (oligosaccharides and total expressed as g/day), fibre (total, soluble and insoluble expressed as g/day), vitamins (A, C, D and E expressed as mg or μg/day) and ORAC (oxygen radical absorbance capacity) index respectively in C, D, SUDD and PD. 

In particular, we assessed the role of some proteins acting as a risk or protective factor for symptomatic diverticular disease, evaluating the consumption of unprocessed red meat, processed red meat (ham/speck/bresaola, cured meats), white meat, fish, cheese (aged cheeses, fresh cheeses) and eggs. On the basis of frequency intake, we considered low, normal or high consumption for each food following these criteria: (i) for processed, unprocessed red meat and cheese, low consumption (as ‘rarely/never’), normal consumption (as ‘more than once a month’, ‘once a week’) and high consumption (as ‘more than once a week’, ‘once a day’, ‘more than once a day’); (ii) for white meat and fish, low consumption (as ‘rarely/never’, ‘more than once a month’, ‘once a week’), normal consumption (as ‘more than once a week’) and high consumption (as ‘once a day’, ‘more than once a day’); for eggs, low consumption (as ‘rarely/never’, ‘more than once a month’), normal consumption (as ‘once a week’, ‘more than once a week’) and high consumption (as ‘once a day’, ‘more than once a day’).

We excluded study participants who reported implausible energy intake (<800 or >4200 kcal/day). 

### 2.3. Statistical Analysis

Data are showed as counts and percentages for the categorical variables and mean and standard deviation (SD) for the continuous variables. The categorical variables were compared using the Chi-squared or Fisher’s exact tests as appropriate. Continuous variables were compared using ANOVA, corrected for multiple comparisons by the Bonferroni procedure. *p* values < 0.05 were considered statistically significant.

## 3. Results

No patient was excluded for reporting implausible energy. Baseline characteristics of all subjects (*n* = 705) are summarized in Table 1. No significant differences between C, D, SUDD and PD patients were found regarding age, gender or BMI. 

**Figure 1:**
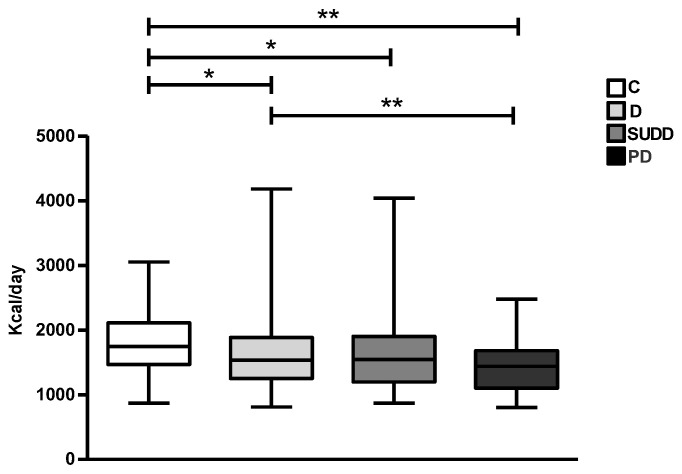
Average daily caloric intake in all groups. C, Controls; D, Diverticulosis; SUDD, Symptomatic Uncomplicated Diverticular Disease; PD, Previous Diverticulitis. * *p* < 0.05; ** *p* < 0.01 (*n* = 705).

The nutritional analysis revealed that daily kcal intake was significantly lower in patients with D, SUDD and PD than C (1640.1 ± 552.8, 1594.6 ± 503.8 and 1439 ± 413.1 vs. 1785.8 ± 451 kcal/day, respectively; *p* < 0.05, *p* < 0.05 and *p* < 0.01) (Figure 1). In addition, patients with PD consumed significantly fewer kcal than patients with D (1439 ± 413.1 vs. 1640.1 ± 552.8 kcal/day; *p* < 0.01) (Figure 1). 

**Figure 2:**
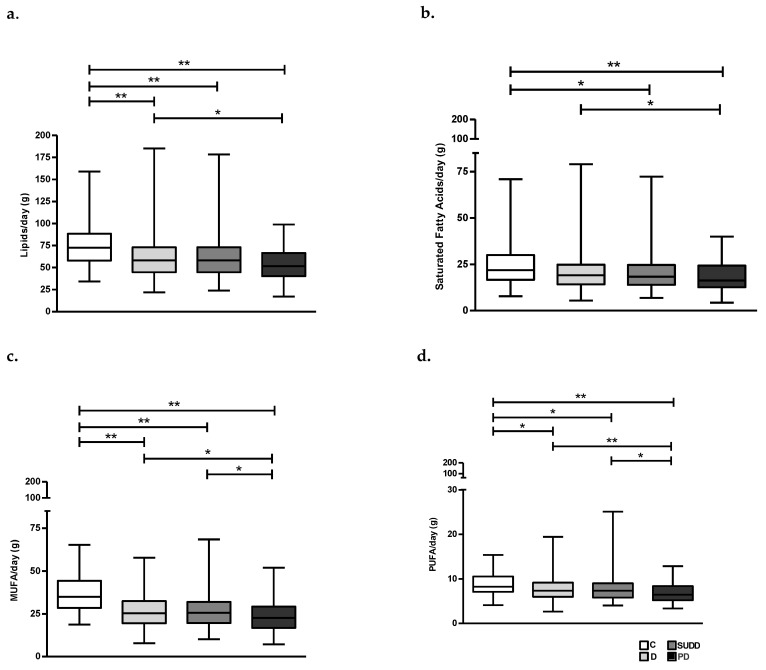
Average daily intake of lipids (**a**), Saturated Fatty Acids (**b**), Monounsaturated Fatty Acids (**c**) and Polyunsaturated Fatty Acids (**d**) in all groups. C, Controls; D, Diverticulosis; SUDD, Symptomatic Uncomplicated Diverticular Disease; PD, Previous Diverticulitis. MUFA, Monounsaturated Fatty Acids; PUFA, Polyunsaturated Fatty Acids. * *p* < 0.05; ** *p* < 0.01 (*n* = 705).

Qualitative macronutrient analysis showed that total daily intake of lipids was also significantly higher in C compared to patients with D, SUDD and PD (62.5 ± 26.9, 61.4 ± 23.7 and 54.2 ± 18.5 vs. 75.2 ± 22.2 g/day, respectively; *p* < 0.01) (Figure 2a) and in patients with D compared to PD (62.5 ± 26.9 vs. 54.2 ± 18.5 g/day; *p* < 0.05) (Figure 2a). For both saturated and unsaturated fats (MUFA and PUFA) the daily average consumption seemed to decrease gradually and progressively from C to PD (Figure 2b–d). 

**Figure 3:**
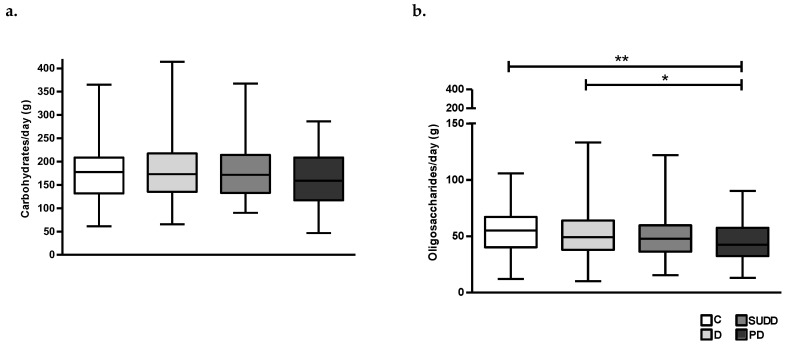
Average daily intake of total carbohydrates (**a**) and oligosaccharides (**b**) in all groups. C, Controls; D, Diverticulosis; SUDD, Symptomatic Uncomplicated Diverticular Disease; PD, Previous Diverticulitis. * *p* < 0.05; ** *p* < 0.01 (*n* = 705).

Total carbohydrate intake was not significantly different between groups; however a deeper analysis showed that patients with PD consumed less oligosaccharides than patients with D and C (52 ± 20.4 and 55 ± 18.9 vs. 45.4 ± 16.2 g/day, respectively; *p* < 0.05 and *p* < 0.01) (Figure 3a,b).

**Figure 4:**
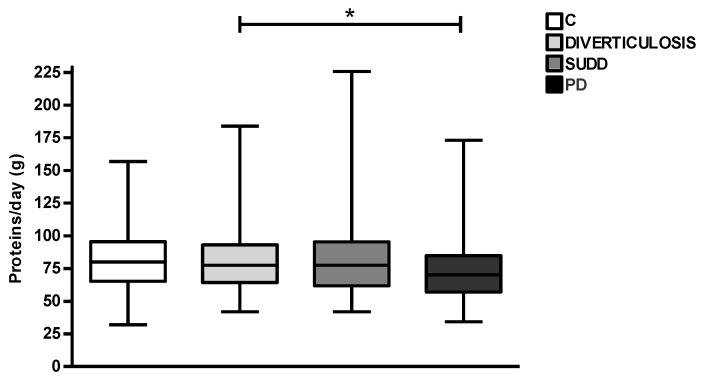
Average daily protein intake in all groups. C, Controls; D, Diverticulosis; SUDD, Symptomatic Uncomplicated Diverticular Disease; PD, Previous Diverticulitis. * *p* < 0.05 (*n* = 705).

Regarding total protein consumption, a significantly lower daily intake was observed in patients with PD than patients with D (72.5 ± 22.4 vs. 82.3 ± 27.2 g/day; *p* < 0.05) (Figure 4). In particular, on the basis of intake frequency, we analysed the consumption of unprocessed and processed red meat, white meat, fish, eggs and cheese (Table 2), finding that the consumption of unprocessed red meat, white meat and eggs was different between groups (*p* = 0.02, *p* < 0.01, *p* = 0.02 respectively).

**Figure 5:**
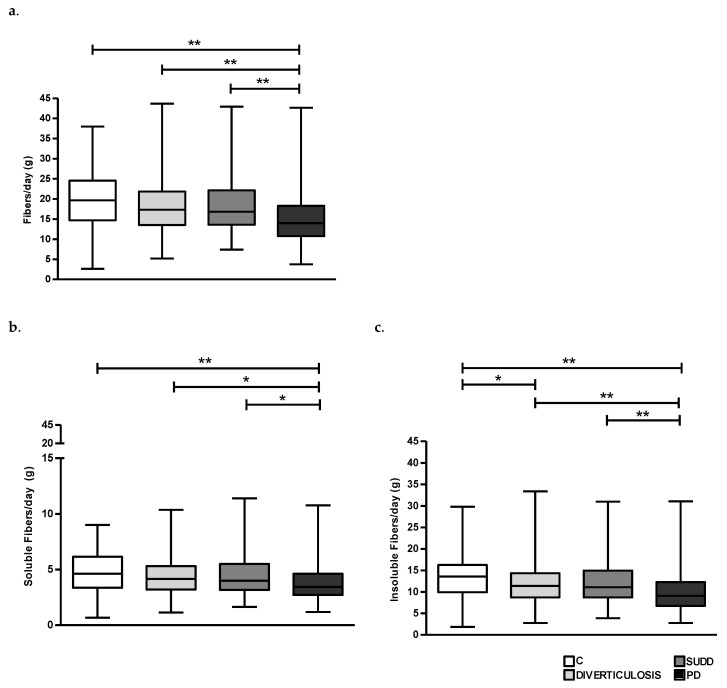
Average daily intake of fibre (**a**), Soluble Fibre (**b**) and Insoluble Fibre (**c**) in all groups. C, Controls; D, Diverticulosis; SUDD, Symptomatic Uncomplicated Diverticular Disease; PD, Previous Diverticulitis. * *p* < 0.05; ** *p* < 0.01 (*n* = 705).

Regarding fibre consumption, we found that the overall daily consumption of fibre seemed to be significantly lower in patients with PD compared to patients with SUDD, D and C (18.3 ± 7, 18.1 ± 6.7 and 19.8 ± 6.8 vs. 15.3 ± 6.3 g/day, respectively; *p* < 0.01) (Figure 5a). This difference is detected also when considering both soluble and insoluble fibre intake separately (Figure 5b,c).

**Figure 6:**
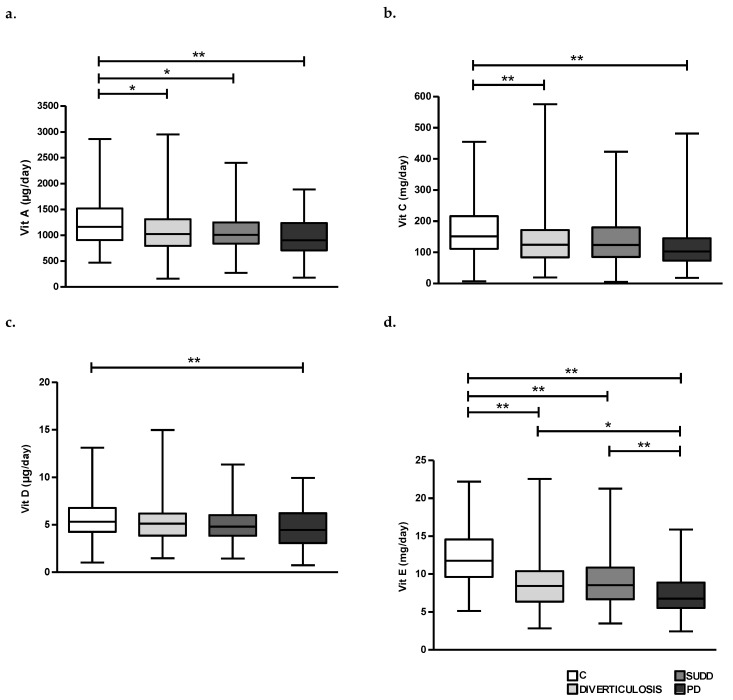
Average daily intake of Vit A (**a**), Vit C (**b**), Vit D (**c**) and Vit E (**d**) in all groups. C, Controls; D, Diverticulosis; SUDD, Symptomatic Uncomplicated Diverticular Disease; PD, Previous Diverticulitis. * *p* < 0.05; ** *p* < 0.01 (*n* = 705).

**Figure 7:**
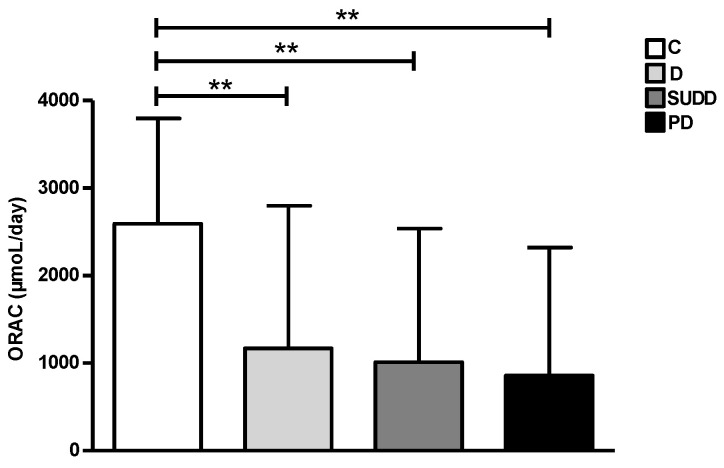
ORAC index in all groups. C, Controls; D, Diverticulosis; SUDD, Symptomatic Uncomplicated Diverticular Disease; PD, Previous Diverticulitis. ** *p* < 0.01 (*n* = 705).

Analysing the amount of dietary vitamins in all groups, we found an overall reduction of all vitamins A, C, D and E in D, SUDD and PD groups compared to C. In particular, daily vitamin A intake seemed to be significantly higher in C group compared to patients with D, SUDD and PD (1090 ± 440.9, 1082 ± 404.1 and 955.1 ± 384 vs. 1231 ± 460.9 µg/day, respectively; *p* < 0.05, *p* < 0.05 and *p* < 0.01) (Figure 6a). Vitamin C intake appeared to be significantly higher in C group than in patients with D and PD (138 ± 79.7 and 118.3 ± 70 vs. 167.4 ± 84 mg/day, respectively; *p* < 0.01) (Figure 6b). Vitamin D intake showed a slight reduction in D and SUDD groups and was significantly lower in PD compared to C (4.7 ± 2.1 vs. 5.6 ± 2.1 µg/day; *p* < 0.01) (Figure 6c). Lastly, the average daily intake of vitamin E appeared to be significantly lower in patients with D, SUDD and PD than C (8.7 ± 3.3, 9.1 ± 3.3 and 7.5 ± 2.9 vs. 12.1 ± 3.3 mg/day, respectively; *p* < 0.01) (Figure 6d). Furthermore, vitamin E intake seemed also to be significantly reduced in patients with PD compared to SUDD and D (8.7 ± 3.3 and 9.1 ± 3.3 vs. 7.5 ± 2.9 mg/day, respectively; *p* < 0.01 and *p* < 0.05) (Figure 6d). According to previous data, ORAC index showed a significant reduction in D, SUDD and PD groups compared to C (1170 ± 1628, 1010 ± 1528 and 859.5 ± 1462 vs. 2591 ± 1203 µmol/day, respectively; *p* < 0.01) (Figure 7). 

## 4. Discussion

Diverticular disease pathogenesis remains poorly defined [18,19], but recently the role of dietary habits emerged as major contributing risk factor for the development of diverticular complications [20]. Population-based studies showed that a high consumption of red meat and a generally Western dietary pattern are risk factors for the development of diverticular complications, mainly acute diverticulitis [10,11], whilst a high consumption of dietary fibre seems to be protective [8,9,21,22,23]. 

However, no data in the different stages of diverticular disease (diverticulosis, SUDD and previous diverticulitis) compared to control subjects in terms of daily calorie, macro- and micronutrient and vitamin intake are available. To assess the whole dietary composition, we used a validated and standardized questionnaire (FFQ), which allows for a global analysis of subjects’ dietary habits [16].

Firstly, we found a gradually significant decreased calorie intake between controls and DD patients, with those with PD consuming fewer calories than C, D and SUDD. Patients affected by inflammatory bowel disease or irritable bowel syndrome, complaining of chronic gastrointestinal symptoms, often change dietary habits, adopting self-imposed food restrictions [24,25]. Similarly, we believe that our DD patients might adopt restrictive eating behaviours and consequently consumed fewer calories because of fear of food. 

The qualitative analysis revealed that DD patients consumed significantly less fats, SFA, MUFA and PUFA, than controls. Dietary fat strongly affects intestinal health by modulating gut microbiota composition and low-grade systemic inflammation. SFA, MUFA and PUFA share important pathways of immune system activation/inhibition with gut microbes, modulating pro-inflammatory profiles. Mechanisms linking dietary fat and gut microbiota are mediated by increased intestinal permeability, systemic endotoxemia and endocannabinoid system [26]. Generally, a high-fat diet and SFA consumption should be avoided, whereas MUFA and PUFA intake should be encouraged in order to modulate gut microbiota and inflammation. Probably, our findings are the result of self-imposed dietary restrictions, similar to the reduced caloric intake previously described. Since our patients are mostly normal weight, we believe that dietary restrictions are not adopted to lose weight. 

Considering overall amount of protein consumption, patients with PD consumed less protein than D. Most interestingly, by looking at the different sources of proteins, we observed that consumption of unprocessed red meat, white meat and eggs was different between groups. In particular, the proportion of DD patients consuming more unprocessed red meat seemed higher compared to C. Our data are in line with those obtained by Cao et al. in their large prospective study [11] and put forward the hypothesis that red meat may influence the risk of DD complications. Red meat may promote chronic low-grade systemic inflammation by an increased level of inflammatory biomarkers and by a direct effect of heme, *N*-nitroso compounds and heterocyclic amines on colon epithelial homeostasis, factors that have been all claimed to play a role in diverticulitis [27,28,29,30]. In addition, we found that the proportion of DD patients consuming more white meat seemed lower than controls. 

As far as total carbohydrate intake, no significant differences were found. However, a more specific analysis showed that PD patients consumed fewer oligosaccharides in comparison to C and D. To our knowledge, this is the first time such data have been reported. It is known that oligosaccharides, like prebiotics, may affect gut microbiota composition enhancing health-associated bacteria growth [31]. We hypothesized that, in our PD patients, the reported lower oligosaccharide consumption might contribute to development of diverticulitis and its relapse. 

Regarding fibre intake, we observed a significantly lower consumption in PD compared to C, D and SUDD respectively, for both soluble and insoluble fibre. In addition, we found also a significantly lower consumption in D compared to C. Our results are in line with previous studies showing that low fibre intake is associated with higher risk of complicated DD, even if no data assessing separately the consumption of soluble and insoluble fibre are available [8,9,21,22]. Crowe et al. in a large UK cohort of middle-aged women found a significantly reduced risk of DD with increased intake of dietary fibre. In detail, they found differences in disease risk by source of fibre, with significant reductions in risk only with intake of fruit and cereal fibre [21]. Mahmood et al. reported that high fruit and vegetable intake may reduce the risk of hospitalisation due to diverticular disease both in women and men, but intake of cereals did not influence the risk [23]. However, in that study, it was not possible to distinguish between diverticulitis, diverticular bleeding and SUDD. Although the benefits related to the consumption of fibre have been extensively reported, traditionally physicians had advised patients to avoid high-residue foods, believing that some foods such as seeds, fruit skins, nuts, etc., may become trapped in diverticula, leading to complications such as diverticulitis or bleeding. However, this concept has been revised. Strate et al. evaluated whether nut, corn or popcorn consumption was associated with diverticulitis and diverticular bleeding, finding an inverse association between nut and popcorn consumption and risk of diverticulitis, but no association was seen between corn consumption and diverticulitis or between nut, corn or popcorn consumption and diverticular bleeding [32]. There are several mechanisms by which fibre intake may influence the risk of diverticulitis, likely by increasing stool bulk, thus reducing intracolonic pressures and stool transit time [33,34,35]. Dietary fibre may also influence gut microbiota composition and metabolic activity and provide a source of short-chain fatty acids, such as butyrate, which have an active role in the regulation of epithelial permeability and mucosal immune activation [36]. We know that a modern Western meat-based diet as compared to a carbohydrate-based diet has significant implications for gut microbiota diversity and its metabolic capabilities [37]. The possible relationship between diet, intestinal microbiota and immuno-inflammatory processes in the gastrointestinal tract is a remarkable topic; however, evidence in DD is scarce. Barbara G et al. showed differences in gut microbiota and colonic immunocytes of DD patients compared with controls. In particular, in patients with DD demonstrated a depletion of microbiota members with anti-inflammatory properties, abundance of mucus-degrading species and an increased number of immune cells in the intestinal mucosa (i.e., macrophages) which was linked to symptoms and inflammation [38]. However, in this study no dietary data have been provided. In the present study, we showed that PD patients, who would benefit the most from high dietary fibre, are those who consumed the least. This dietary habit seems particularly inconvenient in this setting, highlighting the need for targeted nutritional counselling.

Finally, one of our most innovative results is coming from the analysis of dietary vitamin consumption and ORAC index. For the first time, we reported that patients with DD consumed a lower amount of antioxidant vitamins (A, C and E) with a parallel decrease in ORAC index than controls. A recent basic study, conducted on human colonic specimens, showed the presence of oxidative stress-driven myopathy in patients with DD, likely suggesting the possible role of the oxidative balance in this disease [39]. Furthermore, we found a significant lower dietary assumption of vitamin D in PD compared to C. Some conflicting data on the role of serum vitamin D and DD has been reported. In fact, some authors suggested that higher serum levels of vitamin D are associated with a reduced risk of diverticulitis [40]; other data showed that low UV-light exposure, largely influenced by vitamin D, is associated with an increased rate of diverticulitis admissions [12]. On the other hand, other authors do not support this association [41,42]. Although in this study we consider dietary vitamin D and not serum levels, our results supported the protective role of vitamin D against development of diverticulitis. 

This study has some limitations. The main limit is the cross-sectional design that does not allow an assessment of cause-effect relationship between dietary habits and different stages of DD. In addition, information on dietary habits, collected through validated questionnaires, may have been influenced by memory and personal perceptions. Nevertheless, adopting a validated questionnaire such as FFQ is the most feasible method to collect these data. 

## 5. Conclusions

This observational cross-sectional study showed that patients with various stages of DD have different dietary habits compared to control subjects. In particular, DD patients had a lower intake of total daily calories, fats and vitamins than control subjects and PD patients had the lowest consumption of fibre, both soluble and insoluble. The beneficial role of a balanced diet, including high fibre intake, should be strongly emphasized through targeted nutritional counselling, potentially influencing gut microbiota, a key factor in the pathogenesis of DD. Further prospective studies are needed to assess cause-effect relationship between dietary habits and different stages of diverticular disease.

## Figures and Tables

**Table 1:** Baseline characteristics of all subjects. Values are means ± SD unless otherwise indicated. C, Controls; D, Diverticulosis; SUDD, Symptomatic Uncomplicated Diverticular Disease; PD, Previous Diverticulitis. *n* = 705 patients.

	C *n* = 119	D *n* = 344	SUDD *n* = 154	PD *n* = 88	*p* Value
Female sex % (*n*)	52.9 (63)	52.6 (181)	53.2 (82)	52.3 (46)	0.99
Age, y	64.6 ± 9.2	66.4 ± 8.8	66.2 ± 9.6	64.3 ± 11.8	0.11
BMI, kg/m^2^	25.8 ± 3.8	26.6 ± 3.9	26.5 ± 3.8	25.7 ± 3.5	0.06

**Table 2:** Percentage of Low, Normal and High consumption of different sources of proteins in all groups. C, Controls; D, Diverticulosis; SUDD, Symptomatic Uncomplicated Diverticular Disease; PD, Previous Diverticulitis (*n* = 705).

		C *n* = 119	D *n* = 344	SUDD *n* = 154	PD *n* = 88	*p* Value
Unprocessed Red Meat, (%)	Low	15.3	11	5.8	18.2	0.02
	Normal	50.8	44.5	47.4	36.4	
	High	33.9	44.5	46.8	45.5	
Processed Red Meat, (%)	Low	6.8	7.6	7.8	6.8	0.44
	Normal	33.9	24	24.8	31.8	
	High	59.3	68.4	67.3	61.4	
White Meat, (%)	Low	32.2	8.5	26.6	15.9	<0.01
	Normal	61.9	88	69.5	80.7	
	High	5.9	3.5	3.9	3.4	
Fish, (%)	Low	54.2	41.9	39	42	0.06
	Normal	44.9	57.3	60.4	54.5	
	High	0.8	0.9	0.6	3.4	
Eggs, (%)	Low	20.3	30.9	35.3	45.5	0.02
	Normal	77.1	67.3	63.4	54.5	
	High	2.5	1.7	1.3	0	
Cheese, (%)	Low	0.8	6.7	6.5	5.7	0.17
	Normal	17.8	14	15.6	21.6	
	High	81.4	79.2	77.9	72.7	

## Data Availability

Data sharing not applicable.
